# Deleterious effects of maternal ingestion of cocoa upon fetal ductus arteriosus in late pregnancy

**DOI:** 10.3389/fphar.2014.00281

**Published:** 2014-12-22

**Authors:** Paulo Zielinsky, Felipe V. Martignoni, Izabele Vian

**Affiliations:** Fetal Cardiology Unit, Institute of Cardiology of Rio Grande do SulPorto Alegre, Brazil

**Keywords:** Cocoa, polyphenols, anti-inflammatory, antioxidant, fetal ductus arteriosus, ductal constriction, pregnancy

## Abstract

Cocoa powder has twice more antioxidants than red wine and three times more than green tea. Ten percent of its weight is made up of flavonoids. Cocoa has antioxidant and anti-inflammatory effects by downregulating cyclooxigenase-2 receptors expression in the endothelium and enhancing nitric oxide bioavailability. There are evidences that while polyphenols ingestion have cardioprotective effects in the adult, it may have deleterious effect on the fetus if ingested by the mother on the third trimester of pregnancy, causing intrauterine fetal ductus arteriosus (DA) constriction. Polyphenols present in many foods and their anti-inflammatory and antinociceptive activities have been shown to be as or more powerful than those of indomethacin. These effects are dependent on the inhibition of modulation of the arachidonic acid and the synthesis of prostaglandins, especially E-2, which is responsible for fetal DA patency. So, we hypothesized that this same mechanism is responsible for the harmful effect of polyphenol-rich foods, such as cocoa, upon the fetal DA after maternal intake of such substances in the third trimester of pregnancy, thereby rising the perspective of a note of caution for pregnant women diet.

Cocoa powder has twice more antioxidants than red wine and three times more than green tea. Ten percent oh its weight is made up of flavonoids. Its sub-product, dark chocolate, shows in its composition 53.5 mg of catechins in 100 g of chocolate, whereas milk chocolate has 15.9 mg/100 g (Arts et al., 1999).

Biologic activity of polyphenols is related to interference in the inflammatory cascade, with inhibition of prostaglandin synthesis and mediation of nitric oxide synthase 5. Among the discussed effects of cocoa consumption are interferences with vascular function, platelet reactivity and inflammatory processes, suggesting cardioprotective properties of some constituents (Arranz et al., 2013). However, the inflammatory background in disease development, like the starting point of pro-inflammatory cytokine release and metabolite production, is a central target for preventive actions. In line with this, we aim to discuss potential interferences of cocoa antioxidants with central anti-inflammatory mechanisms, by focusing on pathways involved in cyclooxygenase and nitric oxide responses, and its potential side effects.

Cocoa polyphenols modulate nuclear factor kappaB (NF-κB) activity, a protein complex involved in the transduction cascades that play a key role in inflammatory response and carcinogenesis (Sies et al., 2005; Romier-Crouzet et al., 2009). Cocoa polyphenols are potent inhibitors of the kinase activity of mitogen-activated kinase kinase (MEK), and this inhibition subsequently suppresses COX-2 expression in TPA induced inflammation (Heptinstall et al., 2006; Kang et al., 2008). Although, this effect was not observed with cocoa theobromine. MEK is a dual specificity protein kinase that phosphorylates the downstream target ERK on specific tyrosine and threonine residues. Because the expression of COX-2 is primarily regulated by eukaryotic transcription factors such as NF-κB and activator protein (AP)-1, inhibition of AP-1 and/or NF-κB might lead to the suppression of cell transformation through the blocking of COX-2 expression (Hsu et al., 2000; Lee and Lee, 2006; Kang et al., 2008). Stimulation of cells with various tumor promoters an pro-inflammatory substances results in the activation of AP-1 and/or NF-κB by a series of upstream kinases, including those belonging to the mitogen-activated protein (MAP) kinase family. The MAP kinases include extracellular signal-regulated protein kinase (ERK), c-Jun *N*-terminal kinase (JNK), and p38 MAP kinase.

Cocoas (–)-epicatechin and (+)-catechin reduce NF-κB capacity of binding to DNA by preventing the phosphorylation of inhibitor κB (IκB) (Selmi et al., 2006). In a complementary study, Zhang et al. (2006) investigated the effects of procyanidins on COX2 expression on THP-1 cells. NF-κB suppression was accompanied by ERK, JNK, and MAPK inhibition and consequently COX2 expression decreased. These effects occurred by the stabilization of IκB protein to its active site in the NFκB complex (Zhang et al., 2006; Kang et al., 2008).

Furthermore, procyanidin B_2_, a polyphenol with potent anti-inflammatory effect, inhibited inflammasome activation by the inactivation of the NF-κB signaling pathway, the first stage required for the transcription of inflammasome precursors, through the inhibition of p65 nuclear expression and DNA binding, resulting in the transcriptional repression of target genes, such as COX_2_, iNOS and production of IL-6 and NO (Martinez-Micaelo et al., 2014).

Ingestion of cocoa polyphenlos interferes in NO metabolism in various spheres. Procyanidins favor NO synthesis and, thereby, inhibit the formation of superoxide and peroxynitrite (Wollgast and Anklam, 2000). These phenolic compounds cause arterial vasodilation (Corti et al., 2009), decrease infact size (Yamazaki et al., 2014) and increased walking distance in patients with peripheral artery disease by NO mediated mechanisms (Loffredo et al., 2014). Cocoa diminishes arginase activity in endotelial cells, increases L-arginine levels (a substrate for NO production by eNOS) and increases NO metabolites excretion in urine (Andújar et al., 2012). Ramirez-Sanchez et al. (2010) and Moreno-Ulloa et al. (2014), have described that epicatechin acts over eNOS by serine 633 and 1177 phosphorylation and threonine 495 dephosphorylation, which in turn, exerts effect on the intracellular pathway of PI3K, enhancing NO formation. Furthermore, epicatechin causes eNOS uncoupling from caveolin1 (cav-1), by intracellular Ca^2+^ increase, at least, partially mediated by Ca^2+^/CaMKII pathways (Ramirez-Sanchez et al., 2010). NO is an essential mediator of endothelial function, unless it is excessively produced under oxidative stress conditions, it exerts a predominantly antioxidant and anti-inflammatory function. Although, under pathological conditions, the increased NOS activity may not translate into increased NO production. Reduced NO bioavailability through eNOS “uncoupling” from cav-1 is a contributing factor to reduced local NO in various endothelial dysfunctional injuries, like pulmonary hypertension (Zuckerbraun et al., 2011). All isoforms of NOS utilize L-arginine as the substrate, and molecular oxygen and reduced nicotinamide-adenine-dinucleotide phosphate (NADPH) as co-substrates. Flavin adenine dinucleotide (FAD), Flavin mononucleotide (FMN), and (6R-)5,6,7,8-tetrahydro-L-biopterin (BH4) are cofactors of all isozymes (Katusic, 2001). Uncoupling occurs under conditions of reduced BH4 availability where eNOS produces ROS rather than NO. Due to the enhanced oxidative stress seen in many pathological process, an increased degradation of NO by its reaction with O2–⋅ will occur. However, oxidative stress has also been shown to convert eNOS from an NO-producing enzyme to an enzyme that generates O2–⋅. Superoxide anions have been recognized as contributing to vascular dysfunction, through mechanisms including endothelial dysfunction, vascular smooth muscle cell growth, lipid peroxidation, and inflammation (Schewe, 2000; Förstermann and Sessa, 2012).

The effects of flavanols, on the thrid trimester of pregnancy, have not yet been fully described. The human placental function depends on NO and PGI biosynthesis. On the third trimester, there is a relative placentary deficiency of BH4 (Kukor et al., 2000). As pregnancy progresses, oxidative stress in the placenta prepares the uterus for labor (Cindrova-Davies et al., 2007). In this period, NO production may lead to greater risk of nitrative damage.

Polyphenol-rich foods consumption have a positive effect in general health, although deleterious effect on the fetus have been reported if ingested by the mother on the third trimester of pregnancy (Luchese et al., 2003; Zielinsky et al., 2010a,b, 2012a,b, 2013; Zielinsky and Busato, 2013; Bubols et al., 2014). The negative effects on the fetus are possibly due to its anti-inflammatory effect on the ductus arteriosus (DA). Fetal circulation has characteristic features, being morphologically and functionally different from extrauterine circulation. The DA plays a fundamental role in directing the blood flow to fetal inferior body parts. Basically, the DA directs 80–85% of the right ventricular output arising from the superior vena cava, coronary sinus, and a small part from the inferior vena cava to descending aorta. Its histological structure is made up predominantly by a thick muscular layer, differently from the aorta and the pulmonary artery, which increases with gestational age. The fibers have a circumferential orientation, especially at the external layers, facilitating and making effective ductal constriction. These factors may generate lumen alterations which may cause fetal and neonatal complications, such as heart failure, hydrops, neonatal pulmonary hypertension, and even death. Classically, maternal administration of indomethacin and/or other anti-inflammatory drugs interfere in prostaglandins metabolism, causing ductal constriction. In recent years, a growing body of evidence has shown that cocoa, as previously discussed, but also herbs, fruits, nuts, and a wide diversity of substances commonly used in daily diets have definitive effects upon the metabolic pathway of inflammation, with consequent inhibition of prostaglandins synthesis (Zielinsky et al., 2010a). This anti-inflammatory action, when polyphenol-rich substances such as cocoa are ingested during the third trimester of pregnancy, may influence the dynamics of fetal DA flow.

Intrauterine constriction of the fetal DA can be a devastating event that may lead to pulmonary hypertension, heart failure or even perinatal death. Maternal ingestion of anti-inflammatory COX inhibitors are the leading cause of DA constriction, although, up to 65% of cases are considered as of unknown origin (Luchese et al., 2003). We have already shown that after administration of green tea or grape juice to pregnant ewe in the near term the fetuses showed echocardiographic or morphologic characteristics of ductal constriction (Zielinsky et al., 2010a). In another experimental study, the ingestion of green tea as the only source of liquid caused an increase in the flow velocities of the DA, right ventricle hypertrophy and pulmonary hypertension in the echocardiogram of fetal lamb. The histological analysis showed an increase in the avascular media layer, confirming ductal constriction (Zielinsky et al., 2012a). Observational studies in humans also showed that polyphenol rich food ingestion on the third trimester of pregnancy caused ductal contriction (Zielinsky et al., 2010a,b, 2012b; Zielinsky and Busato, 2013) and that the restriction of polyphenols from maternal diet reverted the constrictive effect unto normal flow through the DA even in normal pregnancies (Zielinsky et al., 2012b, 2013). Furthermore, 2 weeks of daily supplementation of polyphenol rich food (total polyphenol of >3100 mg/day) to pregnant ewe caused ductal constriction and decreased plasmatic lipid peroxidation (TBARS) and increased catalase and glutathione peroxidase levels in maternal plasma when compared to normal diet group (Bubols et al., 2014). Even though there was a significant increase of protein carbonylation and lower nitrite/nitrate ratio, PGE2 level were unaltered in maternal blood samples (Bubols et al., 2014; **Table 1**). Specifically in relation to maternal cocoa exposition, in an experimental rodent model of ductal constriction, the administration of one dose of 720 mg/kg of cocoa powder solution through gastric gavage on the last third of pregnancy caused fetal ductal constriction similar to indomethacin 10 mg/kg, but did not alter oxidative stress markers or antioxidant enzymes in the mother or the fetus (submitted).

**Table 1:** Effect of Cocoa, chocolate and polyphenol rich substances and their beneficial effect on the adult or harmful effect on the fetus.

Reference	Substance	Population	Outcome	Overall Effect
Hermann et al. (2006)	Dark chocolate and Liquid cocoa	Healthy humans	Improved endothelial function. Decreased BP	Beneficial
Innes et al. (2003)	Dark chocolate	Healthy humans	Inhibited collagen-induced platelet aggregation	Beneficial
Flammer et al. (2012)	Dark chocolate	Humans with heart failure	Increased FMD	Beneficial
Loffredo et al. (2014)	Dark chocolate	Humans with peripheral arterial disease	Increased FMD, MWD and MWT	Beneficial
Parsaeyan et al. (2014)	Cocoa powder	Humans with Diabetes	Lowered blood cholesterol, triglyceride, LDL-cholesterol, and TNF-α, hs-CRP, IL-6, by Bioinformatics and virtual analysis inhibited COX-2	Beneficial
Morrison et al. (2014)	Epicatechin	Mice with atherosclerosis	Lowers NFkB and atherosclerotic plaque	Beneficial
Lee et al. (2006)	Cocoa polyphenols	Mice and *in vitro*	Inhibited COX2 expression and superoxide formation	Beneficial
Zielinsky et al. (2010b)	Polyphenols rich diet	Healthy pregnant humans	Caused fetal CDA	Harmful
Zielinsky et al. (2013)	Polyphenols rich diet	Healthy pregnant humans	Polyphenol restriction from diet caused fetal ductal dilation	Harmful
Zielinsky et al. (2012b)	Polyphenols rich diet	Pregnant humans with fetal CDA	Polyphenol restriction from diet caused fetal ductal dilation	Harmful
Zielinsky et al. (2007)	Green tea, mate tea, grape juice	Pregnant ewe	Fetal ductus arteriosus constriction	Harmful
Zielinsky et al. (2012a)	Green tea	Pregnant ewe	Fetal ductus arteriosus constriction	Harmful
Bubols et al. (2014)	Polyphenols rich diet	Pregnant ewe	Fetal ductus arteriosus constriction, lowers oxidative stress markers	Harmful

*BP, blood pressure; DAC, ductus arteriosus constriction; FMD, flow mediated dilatation; COX, cyclooxygenase; MWD, mean walking distance; MWT, mean walking time; CDA, constriction of ductus arteriosus*.

In short, the ongoing line of investigation relating maternal exposure to polyphenol-rich substances, including cocoa, in late pregnancy, points toward the perspective of deleterious effects to the fetus, mainly fetal DA constriction, which carries a high risk of neonatal pulmonary hypertension and its potential severe consequences. Additional studies are necessary to establish dose ranges and serum concentrations necessary to determine safety and well being in the human setting.

## Conflict of Interest Statement

The authors declare that the research was conducted in the absence of any commercial or financial relationships that could be construed as a potential conflict of interest.
